# Effectiveness of Implementation of Electronic Malaria Information System as the National Malaria Surveillance System in Thailand

**DOI:** 10.2196/publichealth.5347

**Published:** 2016-05-06

**Authors:** Shaojin Ma, Saranath Lawpoolsri, Ngamphol Soonthornworasiri, Amnat Khamsiriwatchara, Kasemsak Jandee, Komchaluch Taweeseneepitch, Rungrawee Pawarana, Sukanya Jaiklaew, Boonchai Kijsanayotin, Jaranit Kaewkungwal

**Affiliations:** ^1^ Department of Tropical Hygiene (Biomedical and Health Informatics) Faculty of Tropical Medicine Mahidol University Bangkok Thailand; ^2^ Center of Excellence for Biomedical and Public Health Informatics (BIOPHICS) Faculty of Tropical Medicine Mahidol University Bangkok Thailand; ^3^ Thai Health Information Standards Development Center (THIS) Health Systems Research Institute Ministry of Public Health Nonthaburi Thailand

**Keywords:** surveillance system, epidemiology, mixed-methods, evaluation, malaria, eMIS, data quality, public health informatics, Thailand

## Abstract

**Background:**

In moving toward malaria elimination, one strategy is to implement an active surveillance system for effective case management. Thailand has developed and implemented the electronic Malaria Information System (eMIS) capturing individualized electronic records of suspected or confirmed malaria cases.

**Objective:**

The main purpose of this study was to determine how well the eMIS improves the quality of Thailand’s malaria surveillance system. In particular, the focus of the study was to evaluate the effectiveness of the eMIS in terms of the system users’ perception and the system outcomes (ie, quality of data) regarding the management of malaria patients.

**Methods:**

A mixed-methods technique was used with the framework based on system effectiveness attributes: data quality, timeliness, simplicity, acceptability, flexibility, stability, and usefulness. Three methods were utilized: data records review, survey of system users, and in-depth interviews with key stakeholders. From the two highest endemic provinces, paper forms matching electronic records of 4455 noninfected and 784 malaria-infected cases were reviewed. Web-based anonymous questionnaires were distributed to all 129 eMIS data entry staff throughout Thailand, and semistructured interviews were conducted with 12 management-level officers.

**Results:**

The eMIS is well accepted by system users at both management and operational levels. The data quality has enabled malaria personnel to perform more effective prevention and control activities. There is evidence of practices resulting in inconsistencies and logical errors in data reporting. Critical data elements were mostly completed, except for a few related to certain dates and area classifications. Timeliness in reporting a case to the system was acceptable with a delay of 3-4 days. The evaluation of quantitative and qualitative data confirmed that the eMIS has high levels of simplicity, acceptability, stability, and flexibility.

**Conclusions:**

Overall, the system implemented has achieved its objective. The results of the study suggested that the eMIS helps improve the quality of Thailand’s malaria surveillance system. As the national malaria surveillance system, the eMIS’s functionalities have provided the malaria staff working at the point of care with close-to-real-time case management data quality, covering case detection, case investigation, drug compliance, and follow-up visits. Such features has led to an improvement in the quality of the malaria control program; the government officials now have quicker access to both individual and aggregated data to promptly react to possible outbreak. The eMIS thus plays one of the key roles in moving toward the national goal of malaria elimination by the next decade.

## Introduction

Malaria transmission occurs in all six World Health Organization regions. An estimated 3.3 billion people are at risk of malaria infection worldwide, and 1.2 billion are at high risk (>1 in 1000 chance of contracting malaria in a year). In 2013, there were 33,302 confirmed cases in Thailand, with 37 confirmed deaths [1]. Although Thailand is on track to achieving a 50%-75% decrease in case incidence by 2016, the country still faces the challenge of drug resistance, particularly regarding malaria from *Plasmodium falciparum*, to artemisinin treatments [1]. The Thai Ministry of Public Health has introduced several intensive malaria prevention and control strategies and is moving toward malaria elimination [2]. One proposed strategy concerned the introduction of an active surveillance system for effective case management, and thus the electronic malaria information system (eMIS) was developed and implemented in 2009 [3]. Initially, the eMIS was implemented in 7 provinces alongside the Thai-Cambodia border. However, the system now covers 38 malaria-endemic provinces along the Thai-Cambodia and Thai-Myanmar borders and encompasses 147 data entry units. The system has been transmitting epidemiology data from remote areas since October 2011 [2].

The eMIS was originally developed by the Center of Excellence for Biomedical and Public Health Informatics (BIOPHICS) and Mahidol University via support from the World Health Organization and the Bill and Melinda Gates Foundation. The specific aim was to replace the traditional paper-based malaria reporting system with an active and close-to-real-time electronic reporting system [4]. That is, the main goal of the eMIS is to use electronic records that capture daily information about the malaria case management of each individual patient at the point of care. These records can then be retrieved by higher-level health authorities for use in situation analysis. The data flow via eMIS solves the problem inherent in the original paper record reporting mechanism, where authorities would only obtain aggregated data on a monthly basis. Thus, the time delay meant that it was often too late for making any decision about malaria control. With Global Fund support since 2011, the system has been imbedded into the routine work of malaria control authorities in Thailand. Both Web-based applications and mobile technologies have been integrated into the eMIS to enhance and manage case detection, investigation, and follow-up at point-of-care units [2].

The eMIS is operated and overseen by the Bureau of Vector Borne Disease (BVBD), which falls under the Department of Disease Control within the Thai Ministry of Public Health. This occurs through a network of malaria clinics and malaria posts located in villages in malaria-endemic areas. Between the highest decision-making level at the ministry and the local operating sites, there are regional offices of disease prevention and control, vector-borne disease control center (VBDC), and vector-borne disease control unit (VBDU). Currently, eMIS data are hosted at a secure server located at BIOPHICS, only accessible by authorized system users at the Ministry of Public Health. As the eMIS operates under local BVBD staff, BIOPHICS acts as a system developer and active technical system support for the ministry.

**Figure 1:**
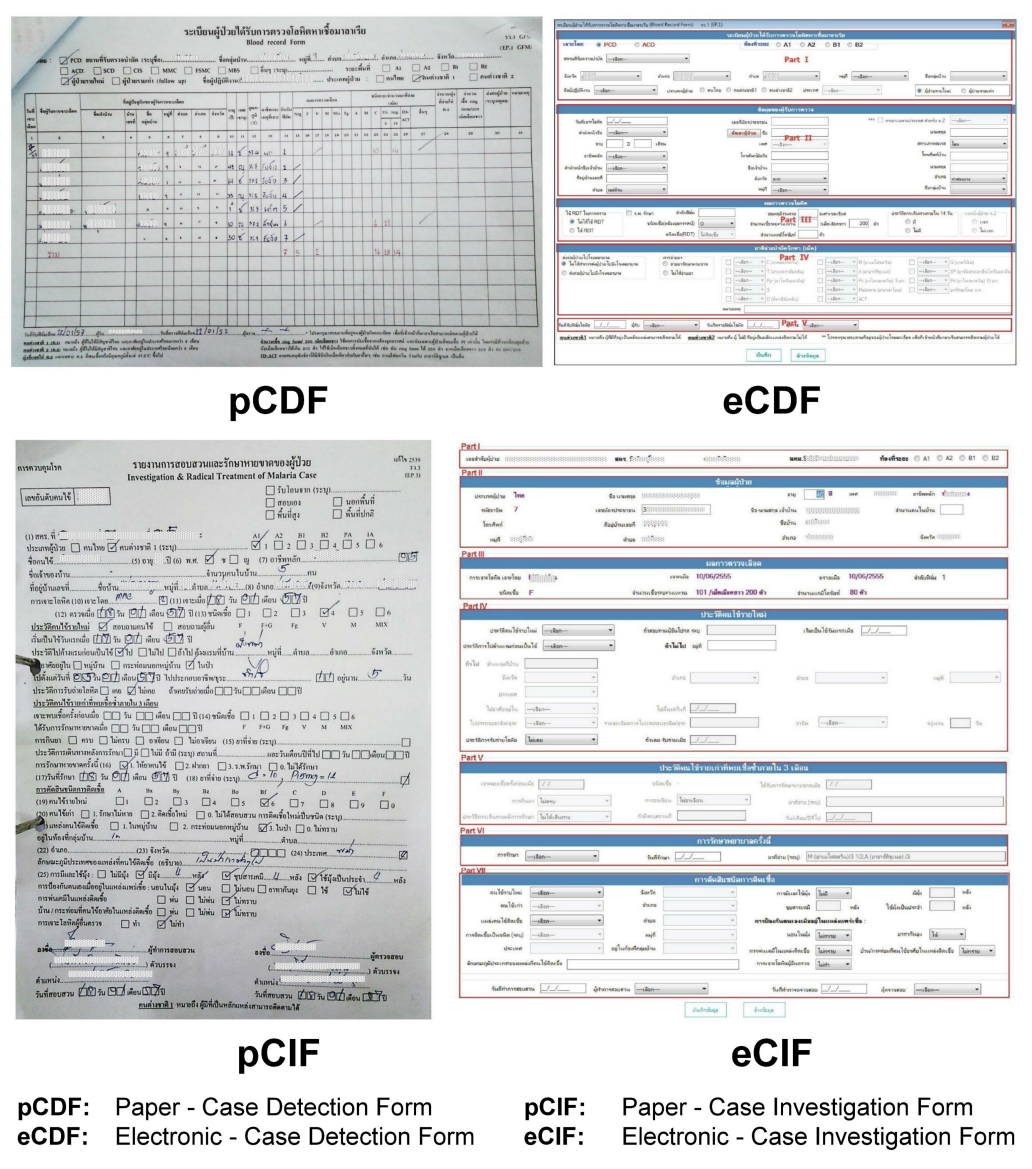
Paper-based forms and electronic data entry screens.

**Figure 2:**
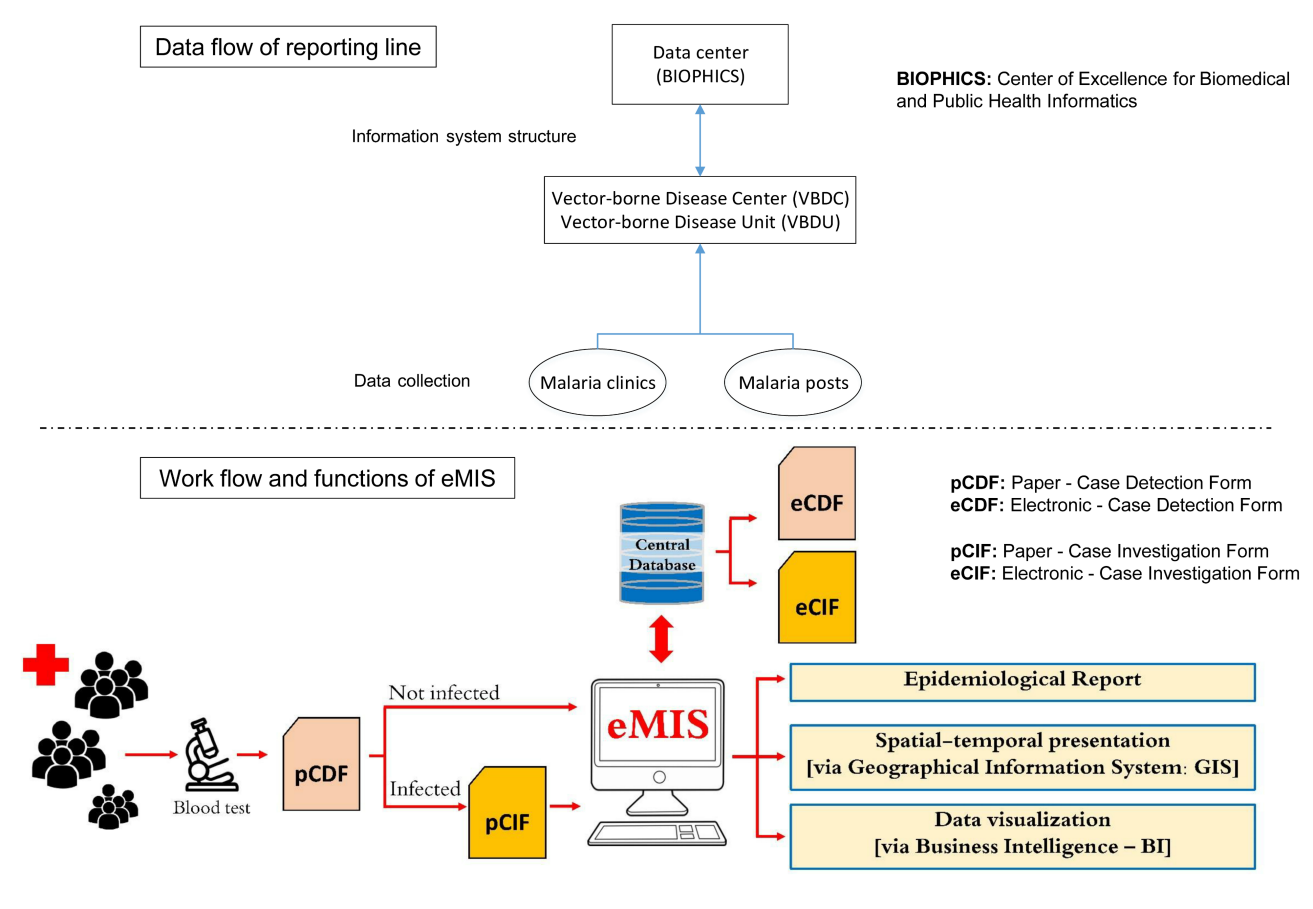
Data flow of the electronic Malaria Information System (eMIS).

The eMIS platform uses offline and online replication technology to enable malaria staff to continue entering data offline when facing an unstable Internet connection. Whenever Internet is available, data can be later synchronized between data entry sites and the central database. Thus, there are some limitations regarding available computer hardware and Internet signal in certain remote areas and/or difficulties in performing simultaneously case management and online data entry. Therefore, each malaria record is captured through a hybrid process in which data are initially collected on paper and subsequently entered into the eMIS via a desktop offline/online client software platform (Figure 1). At point-of-care units (malaria clinics and malaria posts), diagnosis is carried out using a malaria rapid diagnostic test, and standard medical treatment according to national guidelines is immediately administered. A blood film is also collected and sent to the VBDC for archiving and subsequent microscopic confirmation. Both noninfected (negative) and infected (positive) cases are recorded on a “case detection form” (CDF). If a patient’s test result is positive, more information will be collected to record risk factors, signs and symptoms, and the radical malaria treatment provided. Such details are recorded on a “case investigation form” (CIF). Both paper-based CDF and CIF, so-called pCDF and pCIF, are then sent from the malaria point-of-care units in villages in remote areas to VBDC/VBDU where the data are entered either online or offline, and later synchronized with the eMIS. The electronic records of CDF and CIF, so-called eCDF and eCIF, are kept in the eMIS as the national malaria central database, currently located at BIOPHICS’s secure server (Figure 2).

Since its implementation, the eMIS has been evolving; the system has demonstrated its ability to capture essential data from individual malaria cases at local operational units and the data are being used for effective analysis and decision support at upper management levels [4]. Different types of epidemiological reports can be generated and distributed among administrative levels, from the top down to operational levels. Data can be displayed and toggled from tables into graphs via selected variables using business intelligence and geographic information systems for the purpose of creating an effective informatics tool for malaria control and elimination (Figure 2). Informal observations via the periodic training sessions and routine monitoring of the system’s usage show that the eMIS is well accepted by users. However, no formal evaluation of the eMIS has been conducted. Thus, the main research question of this study was to determine how well the eMIS improves the quality of Thailand’s malaria surveillance system. By examining the effectiveness of implementation efforts as evidence-based public health practice [5,6], this study specifically focused on the system users’ perception about the eMIS and the system outcomes in terms of quality of data captured in the eMIS, which were used for management of malaria patients.

## Methods

### Study Design

This is a descriptive study using a mixed-methods evaluation, combining quantitative and qualitative approaches. A number of previous studies have evaluated system innovations using mixed methods and confirmed that multiple evaluation methods can comprehensively identify system and/or electronic record usability, challenges, and specific problems [5,7-9]. In this study, three methods were utilized: data records review, survey of system users (system operating staff), and in-depth interviews with key stakeholders (“advanced” system users and a system developer).

### Study Site and Study Participants/Data Records

Tak and Trat, the two border provinces closest to Myanmar and Cambodia, respectively, were purposively selected as provinces with the highest number of malaria cases along Thailand’s malaria-endemic borders. Source data in the paper-based forms (pCDF and pCIF) versus the electronic data records in the eMIS (eCDF and eCIF) were reviewed and compared. Overall, 4455 pCDFs and 784 pCIFs from the period December 2013 to January 2014 were collected from record books for the two provinces. Similarly, eCDF and eCIF for the same period were extracted from eMIS databases. A Web-based anonymous electronic questionnaire was distributed to all eMIS data entry staff at VBDC/VBDU; of all 129 data entry staff, 128 (99.22%) completed the questionnaire. Semistructured interviews were conducted with management-level users: 1 malaria surveillance officer at the ministry, 12 monitoring and evaluation officers (M&Es) at VBDC/VBDU in Tak and Trat provinces, and 1 information technology (IT) officer at BIOPHICS.

### Data Collection and Data Analysis

There are several methods available for the evaluation of the effectiveness of health care systems, innovation, or tools used for capturing electronic data records [5-8,10,11]. The evidence-based public health practice approach looks at how routinely practices are performed in usual care procedures by analyzing retrospective data from previous site implementation efforts and prospective data from newly adopting sites in terms of implementation and sustainability outcomes [5,6]. Regarding the evaluation of the implementation of a system innovation in terms of its impact and contingency factors, it is necessary to explore changes in workflow and work disruption, data quality, adoption, and user satisfaction [7]. An earlier study on the organizational adoption of systems among family physician practices identified several beneficial aspects and barriers including logistical organization problems, quality of professionals’ clinical decisions, reduction in the cost of managing clinical information, and the barriers faced by system users [8]. A systematic review of the evaluation of surveillance systems highlighted that the approach must be complete; that is, the list of attributes to be assessed should cover not only epidemiological aspects of the evaluation but also social and economic factors. Furthermore, regarding operational factors, a structured process to conduct the evaluation should cover the selection of appropriate attributes and practical methods and tools for their assessment [10]. Thus, in the evaluation of the effectiveness of eMIS, several contextual factors and important attributes and their assessment methods were identified. As an electronic surveillance system for malaria disease, the US Centers for Disease Control and Prevention guidelines for evaluating public health surveillance systems [11] were selected as the evaluation framework for the eMIS. According to the guidelines, the most important attributes for a particular surveillance system and its objectives should be selected. The following attributes are considered key to assess the effectiveness of eMIS: simplicity, flexibility, data quality, acceptability, timeliness, stability, and usefulness. A description of each attribute and its appropriate data collection methods are provided in Table 1.

**Table 1:** Attribute definitions and corresponding data collection approaches.

Attributes	Description^a^	Data collection approaches
Data quality	Completeness (absence of missing values) and validity (absence of errors)	Data record review and semistructured interview
Timeliness	Delay in reporting	Data review
Simplicity	Method of collecting data and time needed to collect data; structure of the system	Structured questionnaire
Acceptability	Willingness of users to use the eMIS^b^	Structured questionnaire
Flexibility	Capacity to cope with new requirements and standards the system follows	Structured questionnaire and semistructured interview
Stability	Downtime of server and response of technical support	Structured questionnaire and semistructured interview
Usefulness	Dissemination of knowledge	Structured questionnaire and semistructured interview

^a^Based on Centers for Disease Control and Prevention *Updated Guidelines for Evaluating Public Health Surveillance Systems* [11], and tailored for the purpose of eMIS [3].

^b^eMIS: electronic Malaria Information System.

Data quality was assessed in terms of the number of records captured using both paper and electronic data collection methods, data completeness or missing data, data validity or consistency between the data in paper and electronic formats, and logical errors or conflicting values among data elements. Timeliness was assessed in terms of delays in data entry from paper data collection forms into electronic records in the eMIS. Both data quality and timeliness were evaluated by comparing the electronic data records in the eMIS (eCDF and eCIF) against the paper data collection forms (pCDF and pCIF). The data from paper-based forms were entered using Microsoft Excel 2013 and double-checked for comparisons with electronic records in the eMIS.

Other surveillance attributes were assessed via a structured questionnaire and semistructured interviews. To fit within the eMIS context, the questionnaire was adapted from a previous study that measured similar attributes concerning data quality in a surveillance system [12]. The questionnaire consisted of choices, dichotomous questions, a 5-point Likert scale, and 1 open-ended question. There were 6 questions asking about the staff practices and perception in using eMIS in terms of time spent, making use of data, and technical problems encountered; 8 items on general impression about the eMIS; 3 questions on eMIS administrative or support team; 4 items on overall thoughts about eMIS; 1 item on eMIS functions that should be improved; and an open question. The anonymous Web-based questionnaire was distributed via Google Form to all data entry sites throughout Thailand; the questionnaire was then responded to by the only one data entry personnel hired by the Ministry of Public Health (one per site). With unknown and unidentifiable responding status, all respondents received a phone call reminder 1 week after the questionnaire was distributed, asking them to complete the questionnaire; this is deemed to be an effective method to achieve a good response rate [13]. Semistructured interviews were conducted with individuals involved with the management and administration of the eMIS. The main purpose of qualitative data collection via in-depth interviews was to gain insightful information or reasons for each attribute measured in the quantitative data analysis. The key interview questions for eMIS officers at the BVBD included issues related to resources used to operate the eMIS, performance of the system and level of usefulness, and opinions on the limitations of the eMIS. The questions for M&E at VBDC included their practices in checking the data quality, the difficulty in managing the system, the use of the data collected by the system, the reports and analysis of the data, and the limitation and suggestion for improvement of the eMIS. The key interview questions for the eMIS technical supporter included their opinions on applying health information standard code sets (ie, Systematized Nomenclature of Medicine -- Clinical Terms [SNOMED CT], *International Classification of Diseases, Tenth Revision* [ICD-10], Health Level Seven [HL7]), the changed or upgraded versions of the eMIS, the evidence of server downtime and time to recover, and the database back up process. The answers from the structured questions were downloaded from Google Forms to be analyzed in Microsoft Excel. The answers to the open-ended question, comments, and interview results were reported verbatim and subsequently reviewed and organized into specific themes related to each attribute shown in the quantitative data analysis section.

### Ethical Considerations

This study was approved by the Ethics Committee of the Faculty of Tropical Medicine, Mahidol University. This study did not involve vulnerable participants. The participants were informed and provided their consent after reading documents explaining the purpose of the study, the participant’s risks and benefits, and the confidentiality and protection of their data. The participants were informed that their answers to the questionnaire were anonymous and would not affect their employment.

## Results

### Data Quality

In general, based on qualitative data collection methods, the data quality of the eMIS was perceived as “good” among management-level users. Officers at the BVBD stated that the data quality of the eMIS was acceptable and better than paper reports, despite no previous evaluation. All M&Es stated that they check the data entered in the eMIS against the paper forms; however, they only check the aggregated case number. One M&E mentioned that the eMIS data were complete while others claimed the data were more accurate than the paper forms.

I think the data quality is acceptable but needs to be improved. But it’s better than paper reports.

Officer at BVBD

The eMIS helps me a lot. The data are more accurate than paper.

M&E

#### Numbers of Initial Paper-Based Forms Versus Electronic Records

In evaluating data quality based on the number of data records being reviewed, the total number of pCDFs did not match eCDF numbers: 38,860 noninfected cases were reported using 4455 pCDFs (several cases were listed on a single paper page) but 41,451 records (one case is one record) were found as eCDFs in the eMIS database. Similarly, fewer infected cases were found in the paper records (one case per single paper page) than those recorded electronically (one case is one record): 781 pCDFs versus 964 eCDFs and 784 pCIFs versus 969 eCIFs.

#### Completeness of Data

Completeness of core data elements (data fields) in the CDFs and CIFs was only checked among infected cases as shown in Table 2. Whereas all elements in the 964 eCDFs showed 100% completeness, among the 781 pCDFs, only the data elements patient name, age, and blood test result were 100% complete. Data were found to be missing for the following elements: date of receiving blood film (186/781, 23.82%; 95% CI 20.83%-26.80%), date of blood test (173/781, 22.15%; 95% CI 19.24%-25.06%), and area classification (266/781, 34.06%; 95% CI 30.74%-37.38%; Table 2). Again, data for the 969 eCIF records were complete, but among the 784 pCIFs the number of missing values for certain elements was high, including area classification (142/784, 18.11%; 95% CI 15.42%-20.81%), followed by infection location (65/784, 8.29%; 95% CI 6.36%-10.22%) and case classification (53/784, 6.76%; 95% CI 5.00%-8.52%).

#### Data Consistency

To evaluate data validity regarding the consistency of the core data elements in the paper and electronic forms, a linkage between the two data entry forms was made. Adapting the data matching method suggested in the literature concerning the assessment of data quality in a cancer registry [14], the linkage of malaria-infected cases between paper and electronic forms was accomplished using patient demographics, blood drawn date, and health care facility location. Such data elements were matched to confirm that each analyzed pair belonged to the same person who was infected at the same time point and was residing in the same location. From the paper and electronic records, 711 pairs of pCDF-eCDF and 719 pairs of pCIF-eCIF were identified and analyzed. The validity of the eMIS data was generally observed as consistent pairs; only a few elements showed significant differences. Among the 711 pCDF-eCDF pairs, discordant pairs were found in the following data elements: area classification (51.88%; 95% CI 47.41%-56.34%), date of receiving blood film (183/711, 25.74%; 95% CI 22.52%-28.95%), and date of blood test (180/711, 25.32%; 95% CI 22.12%-28.51%). Among the 719 pCIF-eCIF pairs, most discordant pairs were found in similar data elements: area classification (260/719, 36.16%; 95% CI 32.65%-39.67%) and infection location (118/719, 16.41%; 95% CI 13.70%-19.12%); for other elements, the discord was less than 10% (Table 3).

**Table 2:** Completeness of core elements.

Core data elements	Total missing	% missing	95% CI	Total missing	% missing	95% CI
**Case detection form**	pCDF^a^(n=781 records)			eCDF^a^(n=964 records)		
Date of form entry	-	-	-	0	0	-
Date of drawing blood	8	1.02	0.32-1.73	0	0	-
Date of receiving blood film	186	23.82	20.83-26.80	0	0	-
Date of blood test	173	22.15	19.24-25.06	0	0	-
Type of blood test	6	0.77	0.16-1.38	0	0	-
Type of patient	4	0.51	0.01-1.01	0	0	-
Area classification	266	34.06	30.74-37.38	0	0	-
Nationality	17	2.18	1.15-3.20	0	0	-
Patient name	0	0	-	0	0	-
Age	0	0	-	0	0	-
Sex	5	0.64	0.08-1.20	0	0	-
Blood test result	0	0	-	0	0	-
Medicine	6	0.77	0.16-1.38	0	0	-
**Case investigation form**	pCIF^a^(n=784 records)			eCIF^a^(n=969 records)		
Date of form entry	-	-	-	0	0	-
Date of drawing blood	1	0.13	0-0.38	0	0	-
Date of blood test	1	0.13	0-0.38	0	0	-
Date of investigation	1	0.13	0-0.38	0	0	-
Area classification	142	18.11	15.42-20.81	0	0	-
Nationality	22	2.81	1.65-3.96	0	0	-
Patient name	0	0	-	0	0	-
Age	3	0.38	0-0.81	0	0	-
Sex	7	0.89	0.23-1.55	0	0	-
Blood test result	1	0.13	0-0.38	0	0	-
Case classification	53	6.76	5.00-8.52	0	0	-
Infection location	65	8.29	6.36-10.22	0	0	-

^a^pCDF: paper case detection form; eCDF: electronic case detection form; pCIF: paper case investigation form; eCIF: electronic case investigation form.

**Table 3:** Agreement between paper-based and electronic data.

Core data elements	No. of discordant pairs	Percentage of disagreement	95% CI
**pCDF^a^-eCDF^a^(n=711 pairs)**			
Date of drawing blood	12	1.69	0.74-2.63
Date of receiving blood film	183	25.74	22.52-28.95
Date of blood test	180	25.32	22.12-28.51
Type of blood test (ACD/PCD)	8	1.13	0.35-1.90
Type of patient (new case/follow-up)	3	0.42	0-0.9
Area classification (A1, A2, B1, B2)	249^b^	51.88	47.41-56.34
Nationality (Thai, M1, M2)	21	2.95	1.71-4.20
Age	7	0.98	0.26-1.71
Sex	7	0.98	0.26-1.71
Blood test result (type of malaria)	2	0.28	0-0.67
Medicine	7	0.98	0.26-1.71
**pCIF^a^-eCIF^a^(n=719 pairs)**			
Area classification (A1, A2, B1, B2)	260	36.16	32.65-39.67
Nationality (Thai, M1, M2)	28	3.89	2.48-5.31
Age	26	3.62	2.25-4.98
Sex	16	2.23	1.15-3.30
Date of drawing blood	10	1.39	0.53-2.25
Date of blood test	9	1.25	0.44-2.06
Blood test result (type of malaria)	13	1.81	0.83-2.78
Date of investigation	60	8.34	6.32-10.37
Case classification	66	9.18	7.07-11.29
Infection location (within village/cottage/forest)	118	16.41	13.7-19.12

^a^ACD: active case detection; pCDF: paper case detection form; eCDF: electronic case detection form; pCIF: paper case investigation form; eCIF: electronic case investigation form; PCD: passive case detection; M1: migrant group 1; M2: migrant group 2.

^b^Denominator varied because some paper-based blood record forms did not include this item.

#### Logical Errors in Data

**Figure 3:**
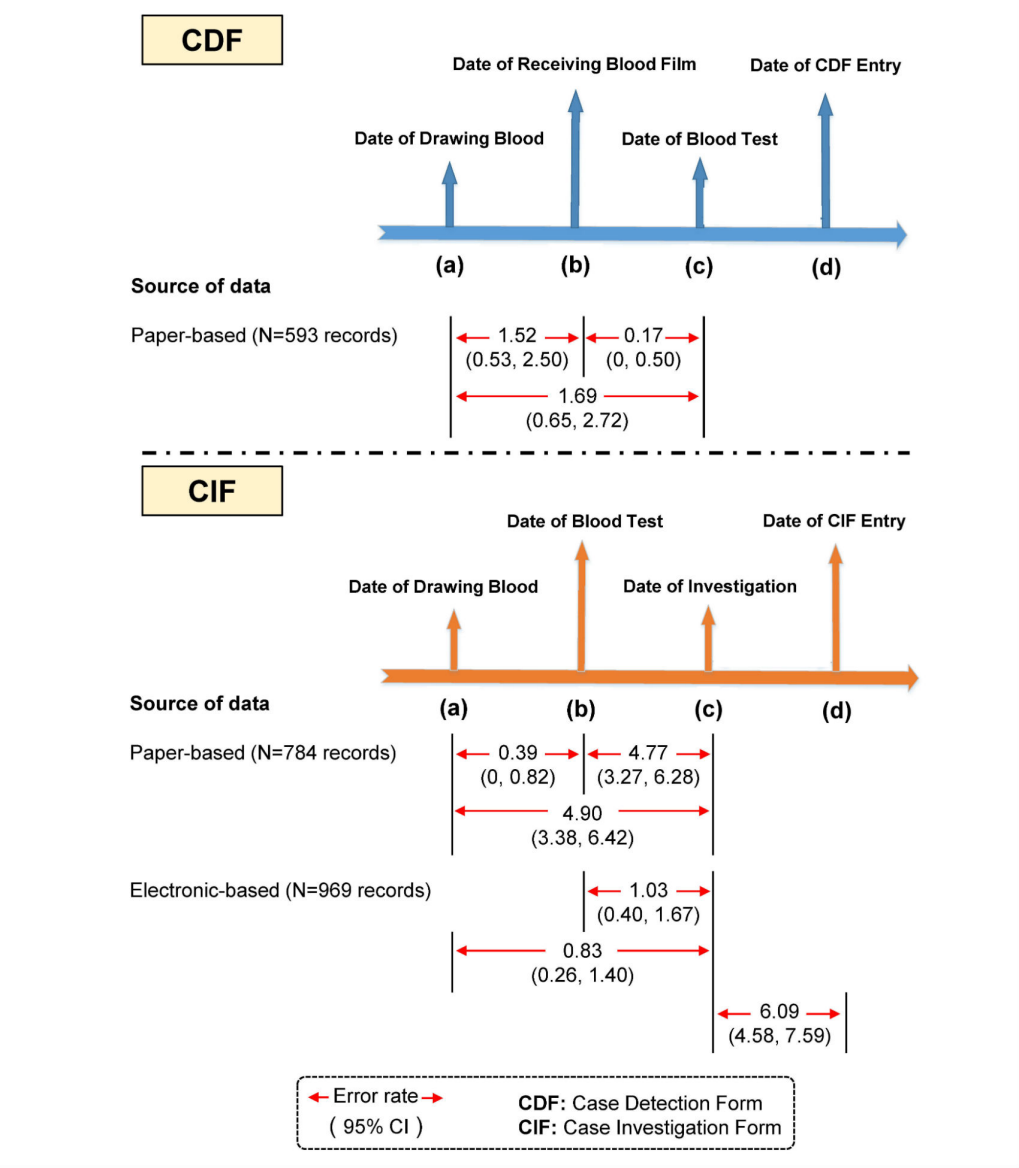
Error rate of date sequence inconsistencies in case detection forms (CDFs) and case investigation forms (CIFs).

A further factor to measure data quality was evaluated: logical errors in the data elements. Logical errors were assessed in two ways: (1) illogical chronological sequence of data capture and reporting dates and (2) discrepancies in reporting malaria prescriptions against national standard guidelines for each type of malaria infection. It should be noted, however, that there were a number of incomplete dates on pCDFs because some infected cases were detected at district or provincial hospitals rather than at the malaria clinics or posts, and hospital health care personnel may not have provided detailed data concerning detection dates (eg, date of receiving blood film, date of blood test) for those infected cases. Thus, complete information regarding the dates of those cases on pCDFs was based on just 593 cases. However, the CIFs were completed and all 784 pCIFs and 969 eCIFs for infected cases were used in the analysis. First, discrepancies in chronological order were checked for different time points (Figure 3). For a CDF, the sequence of dates should be in the following order: (a) date of blood drawn, (b) date of receiving blood film, (c) date of blood test, and (d) date of data entry on the form. A total number of 20 chronological order errors were found in the 593 pCDFs. Illogical date sequences were found among all pCDFs as follows: date (a) > date (b) for 9/593, 1.52% (95% CI 0.53%-2.50%), date (b) > date (c) for 1/593, 0.17% (95% CI 0%-0.50%), and date (a) > date (c) for 10/593, 1.69% (95% CI 0.65%-2.72%). None of the eCDFs showed any discrepancies regarding chronological sequence errors. Similarly, fewer chronological errors were found in the electronic records than on paper forms for case investigation, in which the sequence of dates should follow the following order: (a) date of blood drawn, (b) date of receiving blood film, (c) date of investigation, and (d) date of data entry on the form. Illogical date sequences were found among all 784 pCIFs as follows: date (a) > date (b) for 3/784, 0.39% (95% CI 0%-0.82%), date (b) > date (c) for 37/784, 4.77% (95% CI 3.27%-6.28%), and date (a) > date (c) for 38/784, 4.90% (95% CI 3.38%-6.42%). In the 969 eCIFs, similar discrepancies among dates were also found, with a relatively high number of errors (59 records) found between one type of date order error: date (c) > date (d), at 6.09% (95% CI 4.58%-7.58%; Figure 3).

Another illogical error examined in this study concerned discrepancies in medication prescriptions (type of medicine provided). In Thailand, malaria treatment is regulated and only available at governmental health care facilities for both Thai and non-Thai patients across malaria-endemic areas. Specific standard regimens for each type of malaria infection, mainly *Plasmodium falciparum* and *Plasmodium vivax*, are set as policy according to national guidelines. In this study, prescriptions that differ from the national malaria treatment guidelines were counted as data inconsistencies. Among pCDFs, data inconsistencies in medication prescription were observed at 0.37% (95% CI 0%-1.09%) for *P falciparum* infection and at 0.4% (95% CI 0%-0.94%) for *P vivax* infection. In contrast, among eCDFs, data inconsistencies were recorded at 2.94% (95% CI 1.23%-4.65%) and 1.2% (95% CI 0.32%-2.09%) for prescriptions for *P falciparum* infection and *P vivax* infection, respectively (results not shown).

### Timeliness

In exploring timeliness, the descriptive statistics shown in Table 4 reveal delays in data reporting to the eMIS (ie, data entry date on eCDF) after case detection (ie, blood drawn date on pCDF) and delays in data reporting to the eMIS (ie, data entry date of eCIF) after case investigation (ie, investigation date of pCIF). Note that, in the calculation of delays, records with more than a 200-day delay and negative delays were considered as outliers and/or data entry errors. After excluding such outliers, the mean delay times for case detection and case investigation were 9.34 days (SD 10.63) and 9.14 days (SD 16.27) and median delay times were 4 and 3 days, respectively. From the qualitative information analysis, some M&Es mentioned that paper forms were sent late and therefore they could not enter data in a timely manner.

Some papers arrive late, so we report late.

M&E

**Table 4:** Timeliness analysis.

Reporting delay (days)	Mean (SD)	Median (minimum-maximum)
Delays between date of blood drawn and date of form entry for eCDF^a^(n=963 records^b^)	9.34 (10.64)	4 (0-69)
Delays between date of investigation and date of form entry for eCIF^a^(n=906 records^c^)	9.14 (16.27)	3 (0-134)

^a^eCDF: electronic case detection form; eCIF: electronic case investigation form.

^b^One record with more than a 200-day delay (242 days) was considered an outlier and excluded.

^c^Five records with more than 200 days (369, 372, 372, 366, and 242 days) were considered as outliers and excluded; 58 records with negative delays were excluded, ranging from −1 to −15 days.

### Simplicity

The simplicity of the eMIS refers to both its structure and ease of operation. The eMIS represents a single, vertical, streamlined data entry process. It has a simple and vertical information structure with one central data center at BIOPHICS. All data entry staff report that the time required to enter eMIS data was easy to incorporate into their daily work duties. A total of 85/128, 66.40% of respondents reported spending less than 5 minutes entering into the eCIF the details of 1 infected case (with approximately 80 data elements), and much less time completing 1 CDF via eCDF (42 data elements for a noninfected case and 52 for an infected case). Respondents reported that the time burden for data entry was manageable.

**Figure 4:**
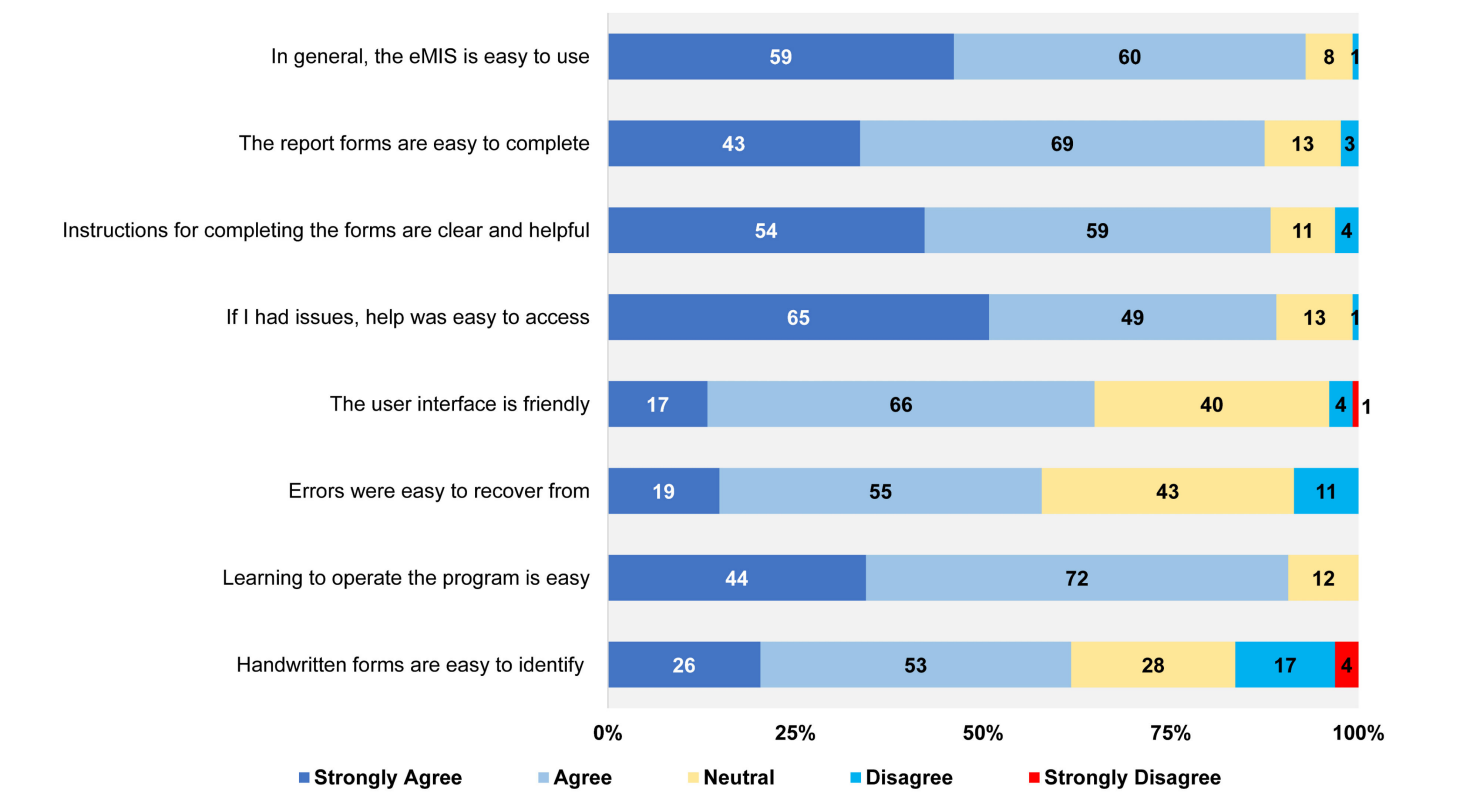
Simplicity of the electronic Malaria Information System (eMIS).

The majority agreed or strongly agreed that the eMIS is easy to use (119/128, 92.97%), the report forms in the eMIS are easy to complete (112/128, 87.5%), instructions for completing the forms are clear and helpful (113/128, 88.28%), help is easy to access (114/128, 89.06%), and that learning to operate the program is easy (116/128, 90.63%). A slightly lower percentage of respondents agreed or strongly agreed that the eMIS has a user-friendly interface (83/128, 64.84%), with 31.25% (40/128) feeling neutral about this statement (Figure 4). Some respondents also commented that a color-labeled data entry screen would improve the system.

The data entry part of the system should be labeled with colors, especially in fields which have been entered.

Data entry staff

The screen for CIF data entry should be separated according to each part of the CIF form. It could use lines or colors to separate each screen for more convenient data entry.

Data entry staff

### Acceptability

Acceptability refers to the willingness of users and organizations to use the system on a daily basis. Initially, the eMIS was implemented in 7 provinces along the Thai-Cambodia border but now includes 38 provinces in Thailand. As of mid-2015, access to the eMIS is expanding to include a number of subdistrict health centers in malaria-endemic provinces.

**Figure 5:**
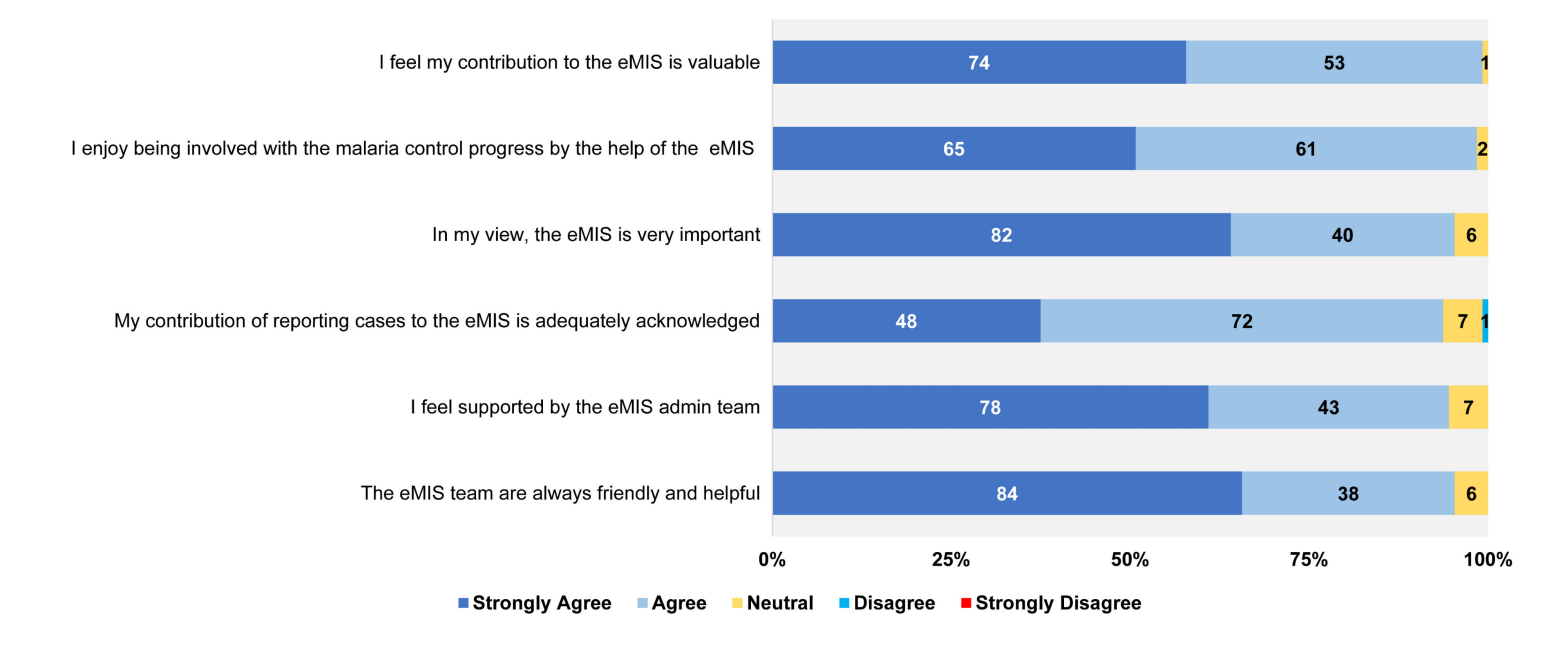
Acceptability of the electronic Malaria Information System (eMIS).

A significant majority of eMIS data entry staff agreed or strongly agreed that their contribution to the eMIS is valuable (127/128, 99.22%), they enjoy being involved with the malaria control progress via the eMIS (126/128, 98.44%), and that their contribution to the reporting of malaria cases to eMIS was adequately acknowledged (120/128, 93.75%). Most (122/128, 95.31%) agreed that the eMIS is very important. Most data entry staff (121/128, 94.53%) felt that they received support from the eMIS support team (Figure 5).

### Flexibility

Flexibility reflects how the system copes with changes. Initially, the eMIS was implemented in 7 provinces as an online system; users were only able to enter data when connected to the Internet. However, because of an unstable Internet connection, the eMIS was changed to include an offline mode. After this big change, eMIS underwent three other main revisions.

We always maintain the core structure and do minor changes according to users’ requirements. Technically, eMIS adapts to change very well.

IT manager of the eMIS

However, the eMIS is not based on any health information standard, such as SNOMED CT, ICD-10, or Health Level Seven. An IT manager mentioned that it would not be difficult to apply some standard code to ensure better data integration in the future.

eMIS only focuses on one disease, malaria, so we didn’t apply any health information standard. But we think it would be good if we applies a standard code in order to integrate our data with the national disease report system in the future.

IT manager of the eMIS

### Stability

Stability reflects the reliability and availability of the eMIS. In all, 100/128, 78.13% of data entry staff reported experiencing some technical problems with the eMIS. The most frequently reported technical problem was data synchronizing, with 87/128, 67.97% of respondents stating they had difficulty synchronizing offline data with the eMIS. This is consistent with interviewed respondents, with 6 M&Es (50%) mentioning the issue of data synchronizing. One M&E specified that data synchronizing was slow, especially at the end of month when many users attempt to upload data at the same time. When asked about technical support for the eMIS, 116/128, 90.63% agreed or strongly agreed the technical issues were dealt with quickly and efficiently.

In terms of reliable resources, the eMIS currently receives funding from the Global Fund and is seeking long-term governmental funding.

At the beginning, eMIS was supported by the Global Fund. Now we plan to use domestic funds. We have both the Global Fund and government funds for staff salaries. In the future, all salaries will be covered by domestic funds.

Officer at BVBD

We have a limited budget for IT after the Global Fund stopped its support. We need a budget for new computers.

M&E

Now the eMIS is very good. But we hope the eMIS will be a long-term program. We need funding to support our IT staff after the Global Fund stops its support.

M&E

The eMIS platform appears stable and performs well with no records of system failure. Data stored in the eMIS is backed up daily to tape media.

The eMIS has never been down since implementation. We have installed and configured virtual machine technology that can perform live migration for the eMIS. Live migration can move a running virtual machine (web and database server) from one physical server to another without any effect to the users.

IT manager at BIOPHICS

By the end of 2016, it is hoped that the management of the eMIS functional hardware and software architecture will be transferred from BIOPHICS to the BVBD at the Ministry of Public Health. One M&E expressed some concern over this change.

In the future, the management of the eMIS will be led by BVBD. My concern is who will take care of the IT problems. Now BIOPHICS do a great job of IT support. And I worry about the salary of IT staff that we will hire for the eMIS in the future.

M&E

### Usefulness

The eMIS is considered a powerful tool and is used by the BVBD in its aim toward eliminating malaria in Thailand. With data from the eMIS, the BVBD is able to change policies, allocate funds, and prioritize malaria elimination activities. The BVBD provides weekly reports and presentations based on eMIS data. An officer at the BVBD reported that the eMIS can influence and guide future research.

We use the results from eMIS to plan elimination. It has helped us to change policy. Before we didn’t have data to indicate how to implement malaria elimination. Now we are involved in subregional elimination. The data from the eMIS help us to target where we are going to eliminate malaria and apply a budget, and also to verify where malaria has been eliminated. eMIS is a powerful tool, without it, I don’t think we can ensure malaria elimination. It’s a key tool for malaria elimination in Thailand to change policy.

Officer at BVBD

At a lower level, all M&Es described the eMIS as being very useful, and considered the eMIS as integral to their routine work. It also provides useful information on malaria patients and tracks malaria patients to ensure effective action. The most commonly reported functions were data analysis, data presentation, and trend analysis. With the eMIS, M&Es are able to track patients, efficiently manage and control any outbreaks, perform campaign and training tasks, and make future plans.

For new cases, we take action quickly and we can predict the management for the next month. With the eMIS, we can see the malaria cases easily and make plans for the future.

M&E

eMIS helps to track patients. We can see the nearby situation and we can get prepared. We can see the real-time situation. We are able to plan how to control before an outbreak and understand the endemic. We can do an analysis for control and prevention. eMIS can quickly transfer data to the VBDU according to the patient’s address. eMIS provides information on the outbreak of disease and accurate patient addresses.

M&E

We compare the malaria cases between different years to see trends in malaria outbreaks. We can track cases and control these cases with well-timed action.

M&E

## Discussion

### eMIS as Thailand’s Malaria Surveillance System

After its development and implementation throughout malaria-endemic areas in Thailand, the eMIS has fully become the national malaria surveillance system. The eMIS appears to be well accepted by system users at both management and operational levels. The reason for its success could be that the design of the eMIS is not overly complex; it still combines original paper-based data collection forms with electronic records. The system flow is practical and can be used as a standard for health care activities in limited-resource settings. Some studies have shown that although paper forms can serve as an important tool for health care personnel in their work, their use can circumvent the intended system design. The following categories describe the benefits of using a paper-based system despite an electronic information system designed for capturing as electronic records: (1) efficiency; (2) knowledge, skill, ease of use; (3) memory; (4) sensorimotor preferences; (5) awareness; (6) task specificity; (7) task complexity; (8) data organization; (9) longitudinal data processes; (10) trust; and (11) security [15]. However, in the eMIS workflow, the paper-based forms are only used as initial data capture at point-of-care units in remote areas. The data are then transferred to the system as individualized electronic data records that can be accessed and utilized throughout the health care system—from point-of-care units to health care management departments. This is in contrast with the original paper-based system in which management could only view aggregated data after some delay, and largely after an outbreak had occurred. In designing any system, it is important to understand the setting so that the technology designers can ensure effective model processes to mitigate any barriers to using eHealth data [16]. Taking into consideration the difficulties of entering an electronic data record directly while caring for malaria patients in remote areas via the eMIS, the system designers decided to use a hybrid of paper-based forms before the introduction of a purely electronic surveillance system. With limited resources and choices in health care settings and a focus on patient-centered services, the system development should aim for a workable system rather than a high-end, perfect solution [17]. The geographically isolated nature of malaria-endemic areas had led to a mixed-design system (combining paper and electronic records) for the eMIS project, representing the most practical and workable solution at the time of system development.

### Practices That Affect Data Quality

In general, data quality was acceptable. The quality of data has provided useful information for malaria personnel to ensure a high level of prevention and control activities. As mentioned above, there were some discrepancies regarding the number of source data forms (pCDF, pCIF) and electronic data records in the eMIS (eCDF, eCIF) for both noninfected and infected cases. There are a number of possible reasons for this result. First, the filing system for the source documents (pCDF, pCIF) was not well organized; some forms may have been misplaced or lost at the time of data collection. Second, as mentioned before, many noninfected cases were captured from the other sources (ie, district or provincial hospitals beyond BVBD primary care units); these were entered directly into the eMIS and thus there were no source documents (pCDF) for such cases. However, almost all infected cases have a source document (pCIF), no matter from which health care facility the data were obtained.

A small number of chronological errors were observed with the pCDF but not for eCDF; however, date errors were found for both pCIF and eCIF. In general, fewer chronological errors were seen in electronic records than on paper-based forms. This type of error might occur because of human error during the filling out of the paper source document and/or data entry into the system. Where the date of the eCIF entry was earlier than the date of investigation, this could be explained as a transcription error in the data entry process. This finding concurs with that suggested in the literature—should be the use of personal digital assistant based data collection or entry system could increase efficiency and reduce data transcription errors for public surveillance data collection in developing countries [18]. However, chronological errors might raise an important data management question: if there were conflicting dates on the paper forms, then why did these not appear in the electronic records when data were entered from the paper source document? In other words, did the data entry staff at VBDC/VBDU enter the same dates as shown on the paper forms? An investigation into this issue revealed that data entry staff usually corrected the dates when entering them into the eMIS because health care staff at point-of-care units can sometimes omit dates on the paper forms. This is particularly true for the date of receiving a blood film and the date of blood test, which match the date of blood drawn. For one electronic health record system [19], it was suggested that data transposition should be as faithful to the original records as possible, given some limitations to the clarity of the originals. However, it has been recommended that logic check programs should be written and integrated into electronic health record systems to reduce the possibility of illogical entry [20].

Another logical check conducted in this study revealed that there were incorrect prescriptions of malaria treatment in both paper-based and electronic data. However, this result should be interpreted with care. This does not mean that the patients received the inappropriate medication; it simply reflects that some patients received treatment that differed from the national standard guidelines. In the eMIS, data elements regarding medication prescriptions are more extensive than the treatment list provided on paper forms. Thus, the conflicting statistics found in this study could occur because some hospital reports did not apply the same malaria treatment as that in the national guidelines.

In this study, a high level of completeness was identified for electronic data records and is consistent with other studies [20-25]. This may due to the fact that there are required fields as well as logic checks across certain fields in the online eMIS. Regarding missing values, high percentages were found for the date of receiving blood film and the date of blood test. As stated before, in real practice, if the date of receiving a blood film and the date of the blood test share the same date as that of drawing blood, then only one date is entered on the paper form for convenience. Interestingly, a high volume of missing values was also observed for “Area classification” for both pCDF and pCIF. Area classification is the term used at the management level to classify the intensity of the malaria burden (eg, A1, A2, B1, and B2 areas) and such definitions change over time. Therefore, data collectors at point-of-care units tend to skip this field when they fill in the paper forms because such information is of no relevance to them. In the electronic records in the eMIS, that data element was completed 100% of the time, probably because of data editing during the data entry process. Data validity check results reveal discordant pairs when comparing data between paper and electronic forms. Such discord was also found in a previous study of the eMIS [26]. In our study, high levels of discord were found for chronological dates, area classification, and infection location. As explained before, the experienced data entry staff at VBDC/VBDU might enter missing values into electronic records based on their own judgment. Thus, the high level of data disagreement might not reflect poor data quality. It is well recognized that data must be complete, consistent, and accurate in any electronic health record system to deliver good health care services. Capturing important data elements is thus critical, but forms are only one part of the procedure to deliver quality health care during a clinician-patient encounter [22]. These results for data quality thus provide evidence-based practices for system improvement, such that (1) paper forms may need to be redesigned, (2) data collectors at the point of care and data entry staff at higher levels should be trained on data quality issues, (3) the need for certain data elements on paper forms (eg, certain dates or classification of areas) should be reconsidered for use at the point of care, (4) to avoid date transcription errors from paper to electronic records, a date-picker function might be the solution to avoid typing errors, and (5) logic validation programming may help reduce human errors.

### System Usability and Suggestions for Improvement

The timeliness analysis indicated that the median time from data captured in paper forms at point-of-care units to data entry as electronic records at VBDC/VBDU was approximately 3-4 days. A previous study on the timeliness of a public health surveillance system [27] indicated that a reporting delay may be the result of several factors as follows: the volume of cases detected at the sites; case follow-up investigations to collect additional case information; system activity due to variable staffing levels; computer system downtime for maintenance, upgrades, or new application development; and data processing routines, such as data validation or error checking. Although timeliness is a key performance measure of public health surveillance systems, it can vary by disease and intended use of data [27]. The timeliness of eMIS is thus considered to be at an acceptable level, as a delay of 3-4 days could still support timely notifications and responses to outbreaks by management personnel. As noted in the Results section, a few cases had outlier values of more than a 200-day delay; this was possibly human errors during the data entry process. A few records showed negative-day gaps; again, this was probably due to a typing error as those records were entered around a change of year, between 2013 and 2014. A logic validation for eMIS might be a solution to reduce human error in the data entry process. Another possible solution is to reduce the time it takes to send paper forms from remote areas to the data entry centers at VBDC or VBDO or to have data entry directly at point-of-care units, which would require a further investment in infrastructure and equipment.

Respondent eMIS users indicated that the system is simple, with the eMIS based on the routine workflow of a vertically structured health care system in Thailand. Most users (both management and operational) accepted the eMIS and were satisfied with its performance; they considered the system easy to incorporate into other work duties. Furthermore, their commitment to be part of the eMIS was high. The M&Es reported being satisfied with the data analysis function and BVBD valued the eMIS as having a key role in the malaria elimination process. Factors influencing the effectiveness of the system have been frequently mentioned in the literature and include trust in quality of the data, motivation of the system users, and outcome expectancy [28-30]. Evaluation via measures that are sensitive to behavior change and feedback strategies is important. The user’s perception is crucial to assure the success of public health systems [28]. Furthermore, differences between “basic” and “advanced” system users can be observed in terms of the expectations of the system’s characteristics and perceived performance outcomes [29]. Similar findings can be seen in other studies about surveillance systems [26,31-34]. The eMIS has different functionalities: it has those that serve operational staff for simple data entry and those that link data for management staff in their data analysis and presentations with mapping and business intelligence tools for decision making. The system’s high participation rates and acceptability among stakeholders show the value of the eMIS.

In assessing the usability of the system, one approach is to define usability as how useful, usable, and satisfying a system is for intended users [35]. A system is useful if it supports the work domain of the users and is independent from the system implementation; a system is usable if it is easy to learn and use and error tolerant; and a system is satisfying if the users have a good subjective impression that the system is useful, usable, and likable. The key characteristics of system usability [35] can be summarized as follows: (1) consistency and standards in design, (2) visibility of system state, (3) matching between the system and the world, (4) minimalist design, (5) minimize memory load, (6) informative feedback, (7) flexibility and customizability, (8) good error messages, (9) prevent use errors, (10) clear closure, (11) undo or reversible actions, (12) use users’ language, (13) users are in control, and (14) help and documentation. Moreover, the usability of a system should be considered whether the system is well adapted to reflect with local priorities in favorable contexts and the use of the collected data [6]. In terms of system flexibility, after its initial implementation there were three major changes; the main system structure was maintained while minor changes were made based on user feedback. As suggested in the literature, the exchange of electronic data records in standard data format can make it easier for data access across institutes, reduce resource waste, and improve the quality of care [36]. However, one drawback concerning the flexibility of the eMIS is that it still does not apply a standard code. The reason for this is that it was designed and used only in a vertically structured malaria control program; however, it could be converted to a standard code should there be such a requirement. Regarding the usefulness of the eMIS, the system has improved the data flow from lower levels to decision-making levels; all interviewed M&Es stated that they frequently used eMIS data to make decisions regarding malaria control (eg, resource allocation and control plan making). Some M&Es sought more information for their data analysis, such as more details for occupation analysis and village-level data analysis. The linkage of data from eMIS to a customizable business intelligence platform has enabled the system users to visualize their own data in the way they want.

Infrastructure is the backbone of every system, and the successful adoption of any eHealth system depends on the infrastructural arrangements [37]. The willingness and interest of system users can be developed and maintained if the system is stable and the users are equipped with appropriate tools and receive regular training. The eMIS has demonstrated great stability: the server has never been down thanks to advanced technical strategies and careful maintenance. Although data synchronization is a common technical problem for data entry staff, it has been resolved by asking users to access the Internet at different times during the end-of-the-month periods when all sites are trying to synchronize data. One system drawback is that eMIS is primarily supported by the Global Fund and requires long-term ongoing funding. Insufficient investment has been proven to be a barrier to health technology in public health systems [32,38]; thus, long-term financial support from the government is essential for eMIS stability.

### Conclusions

Overall, this evaluation, based on data from two malaria-endemic provinces for the period December 2013 to January 2014, has confirmed that eMIS is achieving its objective as an effective platform. The data quality assessment via an intensive look at the data records in the system flow, and the conduct of a Web-based questionnaire survey with all data entry staff throughout Thailand and the in-depth interview with data users from lower level to management level, have suggested that the eMIS helps improve the quality of Thailand’s malaria surveillance system. As the national malaria surveillance system, the eMIS’s functionalities have provided the malaria staff working at the point of care with close-to-real-time case management data quality, covering case detection, case investigation, drug compliance, and follow-up visits. Having access to such information in forms of individual case report, aggregated data in the geographical mapping, and data visualization platform could lead them to respond in a timely manner to the situation in their areas of responsibility. The benefits of eMIS, particularly in assessing the malaria situation, are recognized as exceeding those of the original paper-based reporting system alone. In other words, the eMIS is an information system that supports Thailand’s malaria surveillance. One of the system features that makes the system users satisfied was that it can bring more evidence in the individual practice, for instance by providing electronic reminders about actions to take or treatments to prescribe at point of care (both triggered by previously keyed information). It has also led to an improvement in the quality of the malaria control program because case management is now somewhat standardized and government officials can have quicker access to both individual and aggregated data to promptly react to a possible outbreak. With such features of the system, the eMIS plays one of the key roles in moving toward the national goal of malaria elimination by the next decade.

## Abbreviations

BIOPHICS: Center of Excellence for Biomedical and Public Health Informatics
BVBD: Bureau of Vector Borne Disease
eCDF: electronic case detection form
eCIF: electronic case investigation form
eMIS: electronic Malaria Information System
IT: information technology
M&E: monitoring and evaluation officer
pCDF: paper case detection form
pCIF: paper case investigation form
VBDC: vector-borne disease control center
VBDU: vector-borne disease control unit
VBDO: vector-borne disease control office
